# Cell Type-Specific Imaging of Calcium Signaling in *Arabidopsis thaliana* Seedling Roots Using GCaMP3

**DOI:** 10.3390/ijms21176385

**Published:** 2020-09-02

**Authors:** William Krogman, J. Alan Sparks, Elison B. Blancaflor

**Affiliations:** Noble Research Institute LLC, 2510 Sam Noble Parkway, Ardmore, OK 73401, USA; wlkrogman@noble.org (W.K.); jasparks@noble.org (J.A.S.)

**Keywords:** GCaMP3, Arabidopsis, cell type specificity, calcium response, [Ca^2+^]_cyt_

## Abstract

Cytoplasmic calcium ([Ca^2+^]_cyt_) is a well-characterized second messenger in eukaryotic cells. An elevation in [Ca^2+^]_cyt_ levels is one of the earliest responses in plant cells after exposure to a range of environmental stimuli. Advances in understanding the role of [Ca^2+^]_cyt_ in plant development has been facilitated by the use of genetically-encoded reporters such as GCaMP. Most of these studies have relied on promoters such as *Cauliflower Mosaic Virus* (*35S*) and *Ubiquitin10* (*UBQ10*) to drive expression of *GCaMP* in all cell/tissue types. Plant organs such as roots consist of various cell types that likely exhibit unique [Ca^2+^]_cyt_ responses to exogenous and endogenous signals. However, few studies have addressed this question. Here, we introduce a set of *Arabidopsis thaliana* lines expressing *GCaMP3* in five root cell types including the columella, endodermis, cortex, epidermis, and trichoblasts. We found similarities and differences in the [Ca^2+^]_cyt_ signature among these root cell types when exposed to adenosine tri-phosphate (ATP), glutamate, aluminum, and salt, which are known to trigger [Ca^2+^]_cyt_ increases in root cells. These cell type-targeted GCaMP3 lines provide a new resource that should enable more in depth studies that address how a particular environmental stimulus is linked to specific root developmental pathways via [Ca^2+^]_cyt_.

## 1. Introduction

Calcium (Ca^2+^) plays a vital role as a second messenger system in plant development and stress response. Because Ca^2+^ acts as a stress signal, it is one of the first responses plants have to environmental stimuli. This fast response is achieved through an influx of extracellular Ca^2+^ and an efflux of stored Ca^2+^ from the vacuole, endoplasmic reticulum (ER), and mitochondria that is achieved via a concentration gradient between the intracellular Ca^2+^ stores and the cytoplasm [1]. In non-stimulated conditions, the concentration of Ca^2+^ in the cytoplasm rests around 100 nM [1,2,3]. This low concentration is maintained through H^+^/Ca^2+^ antiporters and Ca^2+^-ATPases that move Ca^2+^ into the apoplast or into intracellular stores [3,4]. Once the response in the cell is initiated, the cell will return to homeostasis by storing excess Ca^2+^ back in the vacuole, ER, or mitochondria through active transport [2], or by pumping it out into extracellular space through gated-ion channels in the plasma membrane.

Once Ca^2+^ enters the cytoplasm in high concentrations, it binds with Ca^2+^-binding proteins to initiate a second messenger cascade [4,5]. Part of this signaling cascade is the activation of Ca^2+^-dependent protein kinases and the regulation of transcription factors to increase tolerance to the stress that caused the initial Ca^2+^ response. Much of our understanding about Ca^2+^ as a second messenger has come from the development of technologies that enable monitoring of free cytosolic Ca^2+^ ([Ca^2+^]_cyt_) changes in living cells. Early on, these technologies consisted of indicator dyes that had to be chemically or physically loaded into the cell, such as Calcium Green or Indo-1. When Ca^2+^ levels in the cell became elevated upon exposing the cell to a specific stimulus, these indicator dyes increased fluorescence [6]. However, use of these indicator dyes in plant cells were often met with technical challenges including uneven dye loading, loss of cell viability after dye loading, and cells failing to take up the dyes [6,7].

Problems with these early indicator dyes were mitigated with the discovery of a suite of fluorescent proteins from jellyfish and other marine organisms [8,9]. Using molecular cloning techniques, selected regions of Ca^2+^-binding proteins could be fused with these fluorescent proteins to generate genetically-encoded Ca^2+^ sensors that change conformation upon Ca^2+^ binding [8]. Ca^2+^ binding induced changes in the conformation of fluorescent protein-based sensors result in enhanced fluorescence emission. One example of a genetically-encoded Ca^2+^ sensor is GCaMP. GCaMP consists of an enhanced green fluorescent protein (eGFP) fused to calmodulin (CaM) and myosin light-chain kinase (M13) [1,10]. GCaMP3 is a modified form of GCaMP that features a circularly permutated enhanced GFP (eGFP) flanked by CaM and M13 that gives low fluorescence in the absence of Ca^2+^, which increases upon reversible binding of Ca^2+^ to the CaM domain [11].

To date, expression of CGaMP and other genetically-encoded Ca^2+^ sensors in plants are driven by constitutive promoters such as *Cauliflower Mosaic Virus 35S (35S)* and *Ubiquitin 10 (UBQ10)* [12,13]. Although plant lines constitutively expressing genetically-encoded Ca^2+^ sensors enabled the functional study of [Ca^2+^]_cyt_ changes across entire plant organs [5,13,14], it is likely that each cell type within the organ is characterized by a unique [Ca^2+^]_cyt_ signature. While [Ca^2+^]_cyt_ changes typically observed in the entire plant organ likely originate from more than one cell type, we lack tools to monitor these changes within specific tissues or cells [15]. To address this need, we generated a set of lines in the model plant *Arabidopsis thaliana*, expressing *GCaMP3* targeted to the columella, endodermis, epidermis, cortex, and trichoblast using cell-type specific promoters [16]. We demonstrate the utility of these new GCaMP3 lines in reporting cell-type [Ca^2+^]_cyt_ changes in roots treated with chemicals previously shown to induce [Ca^2+^]_cyt_ increases in plants.

## 2. Results

### 2.1. Expression of GCaMP3 in Different Root Cell Types of A. thaliana

**Figure 1:**
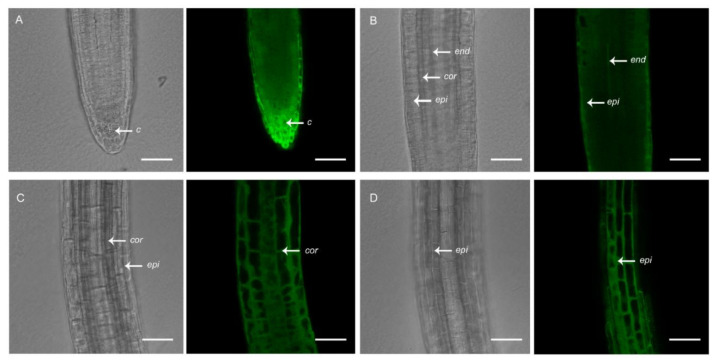
Confocal imaging of primary roots of 5-day-old *A. thaliana* seedlings expressing *UBQ10:GCaMP3*. Representative bright-field and corresponding single-optical section confocal images of the root tip with a focus on the root cap (**A**), meristem (**B**), and elongation zone (**C**,**D**). *c* = columella; *end* = endodermis; *epi* = epidermis; *cor* = cortex. Scale bar: 50 μm.

To the best of our knowledge, plant lines expressing genetically encoded [Ca^2+^]_cyt_ sensors that are available to the scientific community are driven by constitutive promoters. *A. thaliana* lines expressing the *UBQ10:GCaMP3* construct, which we generated and used previously to study [Ca^2+^]_cyt_ oscillations in root hairs [17], is one example. In addition to root hairs, GCaMP3 fluorescence was detected in all cell-types of the primary root of *A. thaliana* expressing the *UBQ10:GCaMP3* construct (Figure 1). Using confocal microscopy, strong fluorescence was observed in cells located at the root surface, such as the peripheral root cap and epidermis (Figure 1A,B,D). Moderate levels of fluorescence could also be detected in the underlying cortex (Figure 1C). However, in cells located deeper within the root, such as the columella and endodermis, fluorescence was obscured by the fluorescence from surface cells (Figure 1A,B).

**Figure 2:**
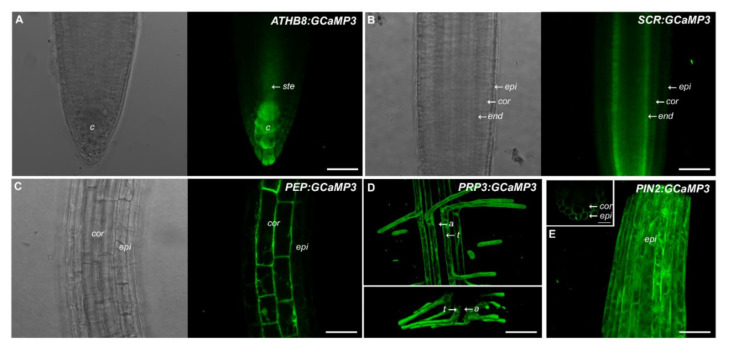
Expression of *GCaMP3* in specific root cell types. Representative bright-field and confocal images of primary roots of 5-day-old *A. thaliana* seedlings expressing *ATHB8:GCaMP3* (**A**), *SCR:GCaMP3* (**B**), and *PEP:GCaMP3* (**C**), respectively. *ATHB8:GCaMP3* is expressed in the columella with some fluorescence visible in the stele. *SCR:GCaMP3* is expressed in the endodermis and *PEP:GCaMP3* is expressed in the cortex. Maximum projection images (top panel in (**D**)) and computer reconstructed transverse section (lower panel in (**D**)) of the root maturation zone of seedlings expressing *PRP3:GCaMP3* show fluorescence confined to the trichoblasts. A maximum projection image of the root elongation zone of seedlings expressing *PIN2:GCaMP3* (**E**). The small box on the upper right corner of panel E shows a computer reconstructed cross-section of the primary root of a *PIN2:GCaMP3*-expressing line. The image was generated from 50 optical sections taken at 0.50 μm intervals. Note that GCaMP3 is predominantly expressed in the epidermis and cortex. *c* = columella; *ste* = stele; *epi* = epidermis; *cor* = cortex; *end* = endodermis; *t* = trichoblast; *a* = atrichoblasts. Scale bar: 50 μm.

This prompted us to generate a set of constructs that drive *GCaMP3* expression in specific root cell types. For this purpose, we used promoters described in the SWELL promoter collection including *ATHB8* (columella), *SCARECROW* (*SCR*, endodermis), *Pin-Formed 2 (PIN2*, epidermis and cortex), *PEP* (cortex), and *Proline rich protein 3(PRP3*, trichoblasts) [16]. For the five constructs, we found that GCaMP3 signal was most prominent in the expected cell types (Figure 2). For example, *ATHB8* and *SCR* promoters drove strong *GCaMP3* expression in the columella and endodermis, respectively (Figure 2A,B). For *ATHB8:GCaMP3*-expressing lines, we also observed weak fluorescence in the peripheral cap cells and the stele. For *SCR:GCaMP3*, fluorescence in the endodermis was observed in all root developmental zones from the meristem and elongation zone, continuing into the maturation zone. Roots of *PEP:GCaMP3*- and *PRP3:GCaMP3*-expressing seedlings also showed fluorescence in the expected cell types (i.e., cortex and trichoblast, respectively) (Figure 2C,D). On the other hand, fluorescence was detected in the epidermis and cortex of *PIN2:GCaMP3*-expressing lines, consistent with observations of Marques-Bueno et al. (2016) [16] (Figure 2E).

### 2.2. Chemical Treatments of Cell-Type Specific GCaMP3 Constructs

Studies on Ca^2+^ signaling in plants over several years have revealed various stimuli that can trigger a rapid elevation in [Ca^2+^]_cyt_ including cold, touch, wounding, hormones, reactive oxygen species, cyclic nucleotides, amino acids and nutrient/ionic stress [5,18,19,20,21,22,23]. We used some of these known [Ca^2+^]_cyt_ triggers to test the responsiveness of our root cell-specific GCaMP3 lines.

**Figure 3:**
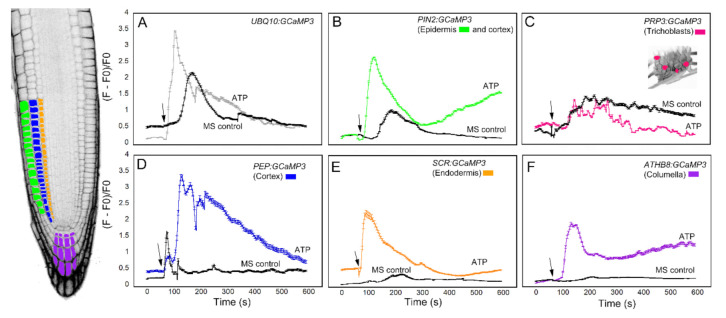
Time course of [Ca^2+^]_cyt_ changes in *A. thaliana* roots after 1 mM ATP application. The leftmost panel shows an inverted fluorescence image of the terminal 300 μm of an *A. thaliana* primary root to illustrate different cells in which *GCaMP3* was expressed. Cell types are color coded with green = epidermis; blue = cortex; orange = endodermis; purple = columella; and the pink cells in the inset in panel C = trichoblast. (**A**–**F**) Quantification of [Ca^2+^]_cyt_ -dependent fluorescence of various lines expressing *GCaMP3* after ATP and solvent control (0.5× MS) application. Black arrows indicate the time of treatment. Plotted values represent the average normalized fluorescence intensity from 3–6 regions of interest (ROI) per line with standard error bars every fifth time point. Fluorescence values were normalized to the lowest fluorescence value.

We first analyzed the response of the GCaMP3 lines to adenosine tri-phosphate (ATP) (Figure 3). In mammalian systems, ATP is a neurotransmitter that is perceived by plasma membrane bound purinergic receptors, and its signaling role is facilitated through increases in [Ca^2+^]_cyt_ [24]. While plants do not have the canonical purinergic receptors found in mammals, they perceive ATP via lectin receptor-like kinases and, similar to mammals, ATP elicits a rapid increase in [Ca^2+^]_cyt_ [25,26,27]. Comparable to previous reports, application of ATP to the elongation zone of roots expressing *UBQ10:GCaMP3* elicited a rapid rise in [Ca^2+^]_cyt_-dependent fluorescence followed by a gradual decline (Figure 3A) [5,25]. To determine if the increase in [Ca^2+^]_cyt_ was due exclusively to ATP, we applied the solvent control solution, which consisted of 0.5× Murashige Skoog (MS) solution without ATP to roots. We found that applying MS solution triggered a rise in [Ca^2+^]_cyt_ that was delayed and lower in amplitude compared to the [Ca^2+^]_cyt_ change caused by ATP (Figure 3A). This observation suggests that under our growing conditions, adding a drop of solvent control solution to the root can induce an increase in [Ca^2+^]_cyt_, which could be the result of a touch or hypoosmotic response [19,20,27,28]. Therefore, in testing the cell-type GCaMP3 lines, we included solvent control applications in all of the experiments so we could tease apart [Ca^2+^]_cyt_ changes due to the desired stimulus from those resulting from a touch or hypoosmotic response.

Upon applying ATP to roots expressing cell-specific GCaMP3 lines, we found similarities and differences in the resulting [Ca^2+^]_cyt_ signatures. For example, the *PIN2:GCaMP3* lines, which expressed *GCaMP3* in the epidermis and cortex (Figure 2), displayed similar [Ca^2+^]_cyt_ signatures as *UBQ10:GCaMP3*-expressing lines in response to ATP and the solvent control solution (Figure 3A,B). This is not surprising given that *GCaMP3* is strongly expressed in both the epidermis and cortex under the control of the *UBQ10* and *PIN2* promoters (Figure 1 and Figure 2). For lines expressing *GCaMP3* in the trichoblasts, application of both ATP and MS control solution triggered a small increase in [Ca^2+^]_cyt_ that had similar patterns (Figure 3C). For lines expressing *GCaMP3* in cells located in the root interior such as the cortex, endodermis, and columella (supplementary video 1), ATP-induced [Ca^2+^]_cyt_ increases resembled the patterns of *UBQ10:GCaMP3* and *PIN2:GCaMP3*-expressing lines. However, the [Ca^2+^]_cyt_ increases triggered by application of MS control solution were not as prominent in these lines when compared to those lines in which *GCaMP3* was expressed in the epidermis (Figure 3D–F).

**Figure 4:**
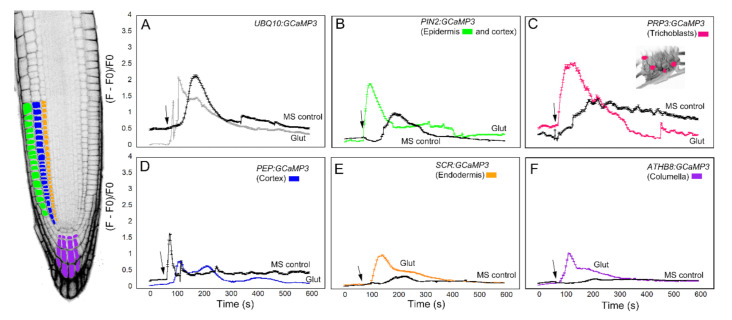
Time course of [Ca^2+^]_cyt_ changes in *A. thaliana* roots after 1 mM glutamate (Glu) application. The leftmost panel shows an inverted fluorescence image of the terminal 300 μm of an *A. thaliana* primary root to illustrate different cell types in which *GCaMP3* was expressed. Cell types are color coded with green = epidermis; blue = cortex; orange = endodermis; purple = columella; and the pink cells in the inset in panel C = trichoblast. (**A**–**F**) Quantification of [Ca^2+^]_cyt_-dependent fluorescence of various lines expressing *GCaMP3* after Glu and solvent control (0.5× MS) application. Black arrows indicate the time of treatment. Plotted values represent the average normalized fluorescence intensity from 3–4 regions of interest (ROI) per line with standard error bars every fifth time point. Fluorescence values were normalized to the lowest fluorescence value.

The next chemical we used to test the cell-specific GCaMP3 lines was glutamic acid (Figure 4). Like ATP, glutamic acid (glutamate) is a neurotransmitter in mammalian cells that can trigger a [Ca^2+^]_cyt_ increase [5,14]. Application of glutamate to the elongation zone of roots expressing the *UBQ10:GCaMP3* construct induced a strong initial [Ca^2+^]_cyt_ increase with a quick return to equilibrium (Figure 4A). The [Ca^2+^]_cyt_ spikes induced by application of MS controls were also observed in this dataset. Compared to glutamate, the onset of [Ca^2+^]_cyt_ increase was delayed in roots treated with MS solution. In this case, however, the [Ca^2+^]_cyt_ spikes triggered by glutamate and the MS control solution were similar in amplitude (Figure 4A). Like the ATP treatment, [Ca^2+^]_cyt_ response of roots expressing *PIN2:GCaMP3* to glutamate was similar to roots expressing *UBQ10:GCaMP3*. Furthermore, [Ca^2+^]_cyt_ changes in the root elongation zone of *PIN2:GCaMP3*-expressing seedlings in response to the MS control solution was delayed, but exhibited a lower amplitude (Figure 4B). Lines expressing *PRP3:GCaMP3* also had a strong initial [Ca^2+^]_cyt_ peak after glutamate treatment with a rapid return to equilibrium that resembled *UBQ10:GCaMP3* and *PIN2:GCaMP3* lines. Like *UBQ10:GCaMP3* and *PIN2:GCaMP3* lines, application of MS control solution elicited a [Ca^2+^]_cyt_ response that was delayed when compared to glutamate exposure (Figure 4C). By contrast, *PEP:GCaMP3*-expressing lines showed a dampened response to glutamate compared to lines in which *GCaMP3* was expressed in the root surface (Figure 4D). Close examination of the time course of [Ca^2+^]_cyt_ signals in the cortex after glutamate application revealed a triphasic response, with two larger peaks and a delayed smaller peak. Surprisingly, the amplitude of glutamate-triggered [Ca^2+^]_cyt_ increases in the cortex was less than that of MS treated roots (Figure 4D). The lines expressing *SCR:GCaMP3* and *ATHB8:GCaMP3* showed monophasic responses to glutamate with a gradual return to equilibrium, similar to lines in which *GCaMP3* was expressed in root surface cells. Furthermore, unlike lines with *GCaMP* expressed in the cortex and root surface cells, the MS control solution response for endodermis- and columella- targeted GCaMP3 had delayed responses and lower amplitudes (Figure 4E,F).

**Figure 5:**
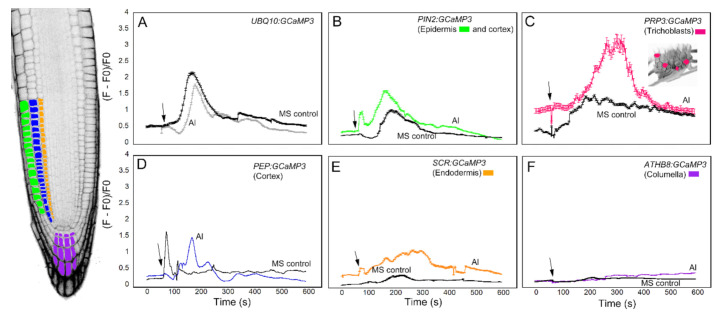
Time course of [Ca^2+^]_cyt_ changes in *A. thaliana* roots after 1 mM aluminum (Al^3+^) chloride application. The leftmost panel shows an inverted fluorescence image of the terminal 300 μm of an *A. thaliana* primary root to illustrate different cell types in which *GCaMP3* was expressed. Cell types are color coded with green = epidermis; blue = cortex; orange = endodermis; purple = columella; and the pink cells in the inset in panel C = trichoblast. (**A**–**F**) Quantification of [Ca^2+^]_cyt_-dependent fluorescence of various lines expressing *GCaMP3* after Al^3+^ and solvent control (0.5× MS) application. Black arrows indicate the time of treatment. Plotted values represent the average normalized fluorescence intensity from 3–7 regions of interest (ROI) per line with standard error bars every fifth time point. Fluorescence values were normalized to the lowest fluorescence value.

The third chemical we tested against the cell-type specific GCaMP3 lines was Al^3+^ in the form of aluminum chloride (Figure 5). Al^3+^ is a toxic tri-valent cation that competes with both Mg^2+^ and Ca^2+^ to increase the activity of Ca^2+^-ATPases while disrupting transport of Ca^2+^ through the plasma membrane [5,29]. Similar to previous treatments, the root elongation zone of seedlings expressing *UBQ10:GCaMP3* showed a monophasic [Ca^2+^]_cyt_ response to Al^3+^ (Figure 5A). The onset of the [Ca^2+^]_cyt_ increase after Al^3+^ treatment was delayed and lower in amplitude compared to ATP, glutamate and solvent control application (Figure 3A, Figure 4A and Figure 5A) [5]. In roots expressing *PIN2:GCaMP3*, Al^3+^ application elicited a biphasic [Ca^2+^]_cyt_ response that consisted of an initial small peak that was quickly followed by a second larger peak, which was broader than the first peak. The onset of the [Ca^2+^]_cyt_ response in *PIN2:GCaMP3*-expressing roots after Al^3+^ treatment occurred earlier than the [Ca^2+^]_cyt_ response triggered by solvent controls (Figure 5B). In roots expressing *PRP3:GCaMP3*, Al^3+^-induced [Ca^2+^]_cyt_ increase was larger in amplitude and had a broader peak compared to those observed in *UBQ10:GCaMP3*- and *PIN2:GCaMP3*-expressing roots (Figure 5C). The [Ca^2+^]_cyt_ increases in roots expressing *GCaMP3* in interior root tissues were less than roots expressing *GCaMP3* in the surface tissues. Among the three lines in which *GCaMP3* was expressed in the interior cell types, the columella-specific GCaMP3 line did not show an Al^3+^-induced [Ca^2+^]_cyt_ peak (Figure 5F). The *PEP:GCaMP3*-expressing lines showed a biphasic [Ca^2+^]_cyt_ response with a distinct first peak followed by a second peak with lower amplitude. Although the amplitude of the [Ca^2+^]_cyt_ peak induced by application of MS solution was similar to the amplitude of the first Al^3+^-triggered [Ca^2+^]_cyt_ peak, the onset of the latter was delayed (Figure 5D). *SCR:GCaMP3*-expressing roots exhibited an Al^3+^-induced [Ca^2+^]_cyt_ response characterized by a broad peak and with a lower amplitude than peaks observed in the root surface- and cortex *GCaMP3*-expressing lines (Figure 5E).

**Figure 6:**
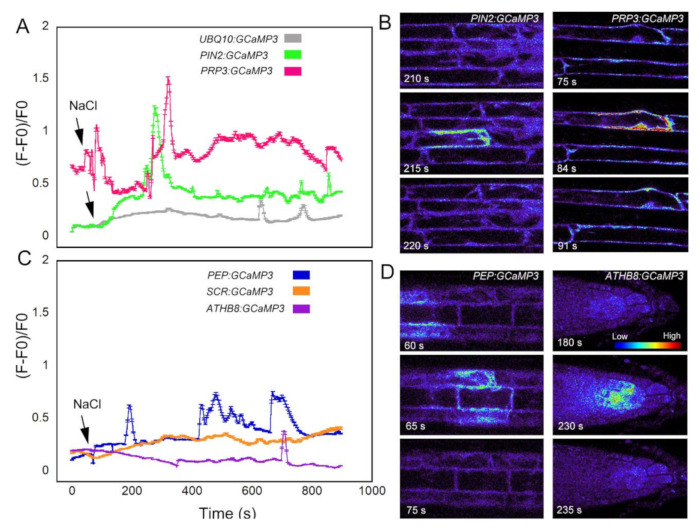
NaCl-triggered [Ca^2+^]_cyt_ transients in cell type-specific GCaMP3 lines. Time course (**A**) and representative heat maps (**B**) of [Ca^2+^]_cyt_ transients in roots expressing *UBQ10:GCaMP3* and root surface cell-targeted GCaMP3 lines. Time course (**C**) and representative heat maps (**D**) of [Ca^2+^]_cyt_ transients in roots where *GCaMP3* is expressed in interior root cell types. Colors of the line graphs in A and C correspond to the cell types shown in preceding figures. The grey line shows the time course of *UBQ10:GCaMP3*-expressing lines. The arrows indicate time of application of sodium chloride. Intensity bar in panel D correspond to high (red) and low (blue) fluorescence with red indicating elevated [Ca^2+^]_cyt_.

The final stimulus we used to test the cell-type specific GCaMP3 lines was salt in the form of NaCl. Na^+^ is used to regulate the voltage equilibrium in cells, but also has direct effects on the efflux of K^+^ into the cytosol [30]. Ca^2+^ is a regulator for the K^+^ efflux channels [30], so disruption of K^+^ into the cytosol may cause variation in the resting concentration of Ca^2+^. In roots expressing the fluorescent-based [Ca^2+^]_cyt_ sensor Yellow Cameleon 3.60 (YC3.60), NaCl treatment resulted in cell-specific [Ca^2+^]_cyt_ transients in the early elongation zone [31]. Consistent with the results of Feng et al. (2018) [31], roots expressing *UBQ10:GCaMP3* exhibited late-onset [Ca^2+^]_cyt_ transients (i.e., >10 min) in cells of the elongation zone (Figure 6A; supplementary video 2). These NaCl-triggered [Ca^2+^]_cyt_ transients were observed in the cell-targeted GCaMP3 lines except for the endodermis-localized GCaMP3 line (Figure 6A–D). In certain lines, the onset of [Ca^2+^]_cyt_ transients occurred much earlier after NaCl application when compared to *UBQ10:GCaMP3*-expressing lines. The onset of NaCl-induced [Ca^2+^]_cyt_ transients was fastest in roots of *PRP3:GCaMP3*-expressing lines followed by *PEP:GCaMP3*-expressing lines. The [Ca^2+^]_cyt_ transients in the columella were only observed several minutes after NaCl application (i.e., 12 min after NaCl application; Figure 6C).

### 2.3. Modification of Growth Conditions and Chemical Application Methods to Mitigate Solvent Control-Induced [Ca^2+^]_cyt_ Transients

As shown in the preceding sections, the 0.5X MS control solution induced [Ca^2+^]_cyt_ changes in *UBQ10:GCaMP3* and lines in which *GCaMP3* was expressed in cells on the root surface and cortex (Figure 3, Figure 4 and Figure 5). Although the MS-induced [Ca^2+^]_cyt_ responses were clearly distinct from ATP, glutamate, and Al^3+^ treatments, we were concerned that the growth conditions in which these first experiments were conducted made roots more sensitive to osmotic changes and/or mechanical perturbation due to the process of adding the solutions. We argued that MS-induced [Ca^2+^]_cyt_ transients could be dampened relative to the chemical applications by: (1) using freshly prepared MS plates for planting seeds and (2) pretreating roots two times with MS solution prior to application of the actual chemical solution. These modifications in our treatment protocols were tested using ATP which typically generated peaks with the highest amplitude.

**Figure 7:**
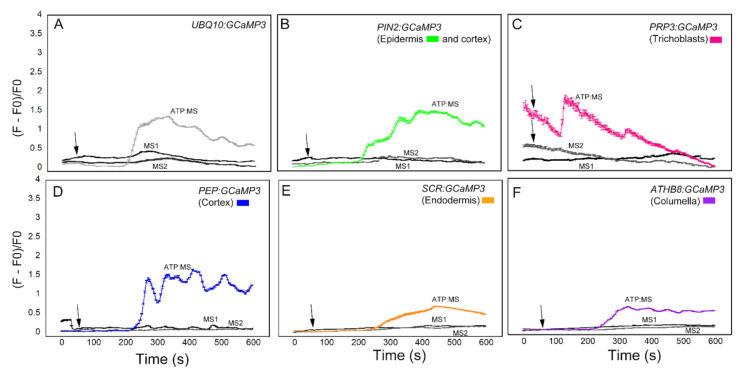
ATP-triggered [Ca^2+^]_cyt_ responses in cell type-specific GCaMP3 lines planted on fresh MS plates and pre-treated twice with MS. MS pretreatment 1 (MS1) time course (black) of [Ca^2+^]_cyt_ in roots show construct treatment with MS control solution. MS pretreatment 2 (MS2) time course (grey) shows construct treatment with a second dose of MS control solution 15 min after first treatment. ATP:MS time course of [Ca^2+^]_cyt_ in roots show cell-type specific response 10 min after MS1 and MS2 treatment. Colors of the line graphs in (**A**–**F**) correspond to the cell types shown in preceding figures with standard error bars every fifth time point. Black arrows indicate the time of application of ATP.

Compared to the datasets shown in Figure 3, Figure 4 and Figure 5, the [Ca^2+^]_cyt_ transients triggered by the MS solution were significantly dampened when fresh plates were used for the experiments (Figure 7). The dampened MS-induced [Ca^2+^]_cyt_ transients were observed in the two sequential MS applications. Pretreatment with MS solution had the added benefit of allowing roots to adapt prior to ATP application (adapted ATP = ATP:MS). Under these modified growth and pretreatment conditions, the ATP-induced [Ca^2+^]_cyt_ increases were lower in amplitude when compared to the [Ca^2+^]_cyt_ increases shown in Figure 3. Despite the lower ATP-induced [Ca^2+^]_cyt_ transients, these [Ca^2+^]_cyt_ responses were better separated from the [Ca^2+^]_cyt_ responses induced by MS solution, particularly in lines expressing *UBQ10:GCaMP3* and those in which *GCaMP3* was expressed in root surface cell types and the cortex (Figure 7). In addition to the dampened response, [Ca^2+^]_cyt_ response to ATP:MS for *UBQ10:GCaMP3* and *PIN2:GCaMP3* lines had broader peaks than those shown in Figure 3. For *PRP3:GCaMP3*-expressing lines, the [Ca^2+^]_cyt_ response was markedly different from the initial ATP treatment (Figure 3C and Figure 7C). Application of ATP after 0.5X MS adaptation caused a strong, monophasic response earlier than either *UBQ10:GCaMP3* or *PIN2:GCaMP3* lines. Lines expressing *PEP:GCaMP3* showed a continued elevated [Ca^2+^]_cyt_ response that oscillated throughout the 10 min time course compared to either *UBQ10:GCaMP3* lines or the previous ATP treatment (Figure 3D and Figure 7D). For lines expressing *SCR:GCaMP3* or *ATHB8:GCaMP3*, the response was similar to previous ATP treatments, but without the strong transient peak (Figure 3E,F and Figure 7E,F). After the cell-type specific lines were adapted to the 0.5× MS control solution, the response time for ATP:MS was initiated later than the previous ATP treatment (Table 1).

To further tease out the differences among *GCaMP3*-expressing lines, we obtained the time elapsed from chemical application to maximum GCaMP3 fluorescence (referred to as reaction time), and analyzed the relationship between chemicals using Analysis of Variance (ANOVA) with Tukey Post-Hoc test (Table 1). Our analysis showed that reaction time was delayed in lines where GCaMP3 was expressed in inner cell types. When comparing the different chemical treatments, ATP, glutamate, and Al^3+^ showed similar responses (except *ATHB8:GCaMP3* to Al^3+^) while adapted ATP:MS showed significantly slower reaction times to both ATP and glutamate (Table 1). However, with such a small sample size, statistical significance may indicate a trend rather than actual significance. NaCl reaction time was not included due to the large variation in responses across each line.

### 2.4. Evaluation of [Ca^2+^]_cyt_ Signatures from Similar Root Development Regions and Cell Types

**Figure 8:**
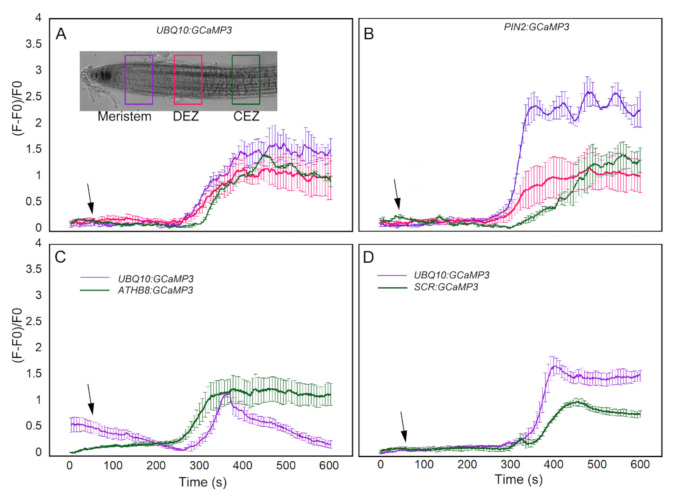
Direct comparison of ATP-induced [Ca^2+^]_cyt_ signatures in equivalent root developmental regions between *UBQ10:GCaMP3* and cell-type specific GCaMP3 lines. Regions of interest (ROIs) were drawn in the meristem, distal elongation zone (DEZ), and central elongation zone (CEZ) in *UBQ10:GCaMP3* (**A**) and *PIN2:GCaMP3* (**B**) roots (inset in A). Comparison of ATP-induced [Ca^2+^]_cyt_ signatures in presumptive columella and endodermis of *UBQ10:GCaMP3* lines with *ATHB8:GCaMP3* (**C**) and *SCR:GCaMP3* (**D**). Black arrows indicate the time of treatment. Plotted values represent the average normalized fluorescence intensity from 3-4 ROI per line with standard error bars every fifth time point. Fluorescence values were normalized to the lowest fluorescence value.

In comparing *UBQ10:GCaMP3* lines with the other cell-type specific GCaMP3 lines, a ROI was selected and the average of normalized fluorescence intensity was plotted. For the most part, the ROI in *UBQ10:GCaMP3* lines was from the root epidermis and the average normalized fluorescence intensity from this ROI was compared with that of individual or groups of cells from the cell-type specific GCaMP3 lines. This process did not enable direct comparisons of [Ca^2+^]_cyt_ signatures between the same cell types or root developmental regions. To address this issue, we conducted another set of imaging experiments using ATP-treated *UBQ10:GCaMP3* and *PIN2:GCaMP3* lines. For these experiments, a more direct comparison was conducted by drawing ROIs in epidermal cells from similar positions along the root longitudinal axis encompassing the meristem, distal elongation zone, and central elongation zone (Figure 8A). In doing so, we found similarities and differences in [Ca^2+^]_cyt_ signatures between *UBQ10:GCaMP3* and *PIN2:GCaMP3* lines. For example, the ATP-induced [Ca^2+^]_cyt_ signatures of *UBQ10:GCaMP3* in all root developmental regions were identical to those in the distal and central elongation zone of *PIN2:GCaMP3* lines. In *PIN2:GCaMP3* lines, however, the ATP-induced [Ca^2+^]_cyt_ signature had a much greater amplitude in the meristem (Figure 8A,B).

We extended our analysis by drawing ROIs in presumptive columella or endodermis of *UBQ10:GCaMP3* and comparing the resulting [Ca^2+^]_cyt_ signatures from these ROIs with those of *ATHB8:GCaMP3* and *SCR:GCaMP3*. For the columella, we found that *ATHB8:GCaMP3* lines displayed an ATP-triggered [Ca^2+^]_cyt_ signature with a sustained peak throughout the 10 min time course. On the other hand, the ATP-induced [Ca^2+^]_cyt_ signature from the presumptive columella region of *UBQ10:GCaMP3* lines was transient (Figure 8C). For the endodermis, the peak of ATP-induced [Ca^2+^]_cyt_ increase was higher in *UBQ10:GCaMP3* when compared to *SCR:GCaMP3* (Figure 8D).

## 3. Discussion

In this paper, we introduced a set of *A. thaliana* lines expressing the intensiometric [Ca^2+^]_cyt_ reporter GCaMP3 targeted to specific root cell types. Roots of these cell type specific GCaMP3 lines responded to chemical treatments such as ATP, glutamate, Al^3+^, and NaCl by exhibiting increases in [Ca^2+^]_cyt_-dependent CGaMP3 fluorescence. Imaging [Ca^2+^]_cyt_ changes in cell types in response to some of these chemicals using lines in which genetically-encoded [Ca^2+^]_cyt_ reporters are expressed ubiquitously have been attempted previously. This was done by drawing a region of interest corresponding to a particular tissue/cell type. For example, it was shown that Al^3+^-induced biphasic [Ca^2+^]_cyt_ signatures in the root elongation zone occurred predominantly in the cortex when compared with the epidermis [5]. While this has proven effective for marking developmental regions along the longitudinal axis of the root [5,27], specifying a region of interest to designate root tissue/cell types along the radial axis for fluorescence-based measurements of [Ca^2+^]_cyt_ can be problematic because this does not guarantee complete separation of signals from adjacent cell types. This problem becomes even more challenging for cell types located in the innermost regions of the root such as the columella and endodermis. Moreover, the ability to consistently draw a region of interest to outline the root cortex requires that the root grew in a particular orientation during the imaging experiments [5]. Here, we documented some of the advantages of the cell type specific GCaMP3 lines by comparing [Ca^2+^]_cyt_ signatures from ROIs marking presumptive columella and endodermis in *UBQ10:GCaMP3* lines with those of *ATHB8:GCaMP3* and *SCR:GCaMP3*. This analysis revealed stark differences in both the amplitude and shape of [Ca^2+^]_cyt_ signatures. Differences were also uncovered when equivalent root developmental regions were compared, particularly with regard to the amplitude of [Ca^2+^]_cyt_ signatures in the meristem (Figure 8). The differences are likely due to contaminating signals from other cell types in *UBQ10:GCaMP3* lines. The use of cell type specific GCaMP3 lines described here could potentially mitigate such problems.

The [Ca^2+^]_cyt_ transients in the cell type specific lines induced by these various chemicals had various patterns when compared with lines in which *GCaMP3* was expressed ubiquitously. A similarity in patterns occurred within the root surface cells’ responses to the different chemical treatments and again within the root inner cell types. Lines in which GCaMP3 was expressed in innermost root cell types such as the columella and endodermis generally had slower response times when compared to lines with GCaMP3 expressed in root surface cells. The delayed response times in lines with GCaMP3 expressed in the columella and endodermis was observed in all chemical treatments (Table 1; Figure 6). One could argue that because of the innermost location of the columella and endodermis, the delay in the response times might be due to the longer time it takes for the chemical to make contact with the target cells. The fact that columella and endodermis exhibit minimal or no [Ca^2+^]_cyt_ increase after application of 0.5X MS supports this possibility. An alternative hypothesis is that the delayed [Ca^2+^]_cyt_ response in the innermost cell types is due to an inward moving [Ca^2+^]_cyt_ wave, which is not easily revealed when GCaMP3 is expressed ubiquitously or on the root surface. From our data, these inward-directed [Ca^2+^]_cyt_ waves appear to be most prominent in ATP- and glutamate-treated roots (Figure 3, Figure 4 and Figure 7). The GCaMP3 lines described here could present a significant tool for better understanding the radial direction of [Ca^2+^]_cyt_ waves in roots. A meaningful set of experiments with GCaMP3 lines expressed in innermost cell types will be to locally apply a small volume of the chemical stimulus to the root epidermis.

The line that was most similar to *UBQ10:GCaMP3* with regard to chemical-induced [Ca^2+^]_cyt_ transients was *PIN2:GCaMP3*. This was not surprising given that the *PIN2* promoter drives *GCaMP3* expression in root epidermal cells, which is the cell type that is readily imaged in *UBQ10:GCaMP3* lines due to its location on the root surface. With the *PEP:GCaMP3* lines described here, we were able observe biphasic, triphasic and oscillatory [Ca^2+^]_cyt_ signatures after Al^3+^ treatment that were reminiscent of some of the observations made by Rincon-Zachary et al. (2010) [5] (Figure 5). The cortex- and trichoblast-targeted GCaMP3 lines also revealed an earlier onset of NaCl-induced [Ca^2+^]_cyt_ transients when compared to *UBQ10:GCaMP3* lines (Figure 6). It has been shown that [Ca^2+^]_cyt_ transients in roots exposed to NaCl is part of the cellular machinery that prevents root cells from rupturing under salinity stress. The plasma membrane-localized receptor-like kinase, Feronia (FER), is required for root growth recovery after encountering high salinity conditions [31]. Because *fer* mutants displayed a high incidence of cortical cell rupture when compared to wild type after exposure to NaCl, it is tempting to speculate that earlier onset of [Ca^2+^]_cyt_ transients in the cortex revealed by the *PEP:GCaMP3*-expressing lines could be a mechanism to maintain root growth integrity. The cell type GCaMP3 lines described here should be useful in testing this hypothesis.

While testing the various cell type specific GCaMP3 lines, we encountered a number of technical challenges brought about by our seedling growth conditions. In several experiments, we found that applying the 0.5X MS control solution alone could trigger a rise in [Ca^2+^]_cyt_. The MS only-induced [Ca^2+^]_cyt_ transients were most prominent in the *UBQ10:GCaMP3*-expressing lines and those in which *GCaMP3* was expressed in root surface cell types (e.g., *PIN2:GCaMP3* and *PRP3:GCaMP3*). The [Ca^2+^]_cyt_ transients observed after MS treatment were reminiscent of touch-induced [Ca^2+^]_cyt_ responses [20], and recent studies on ATP-induced [Ca^2+^]_cyt_ signaling [27], suggesting that we might have been eliciting a touch response as the various solutions were added to the root. In support of this hypothesis, our method for root [Ca^2+^]_cyt_ imaging involved planting seed of the reporter lines on a thin layer of agarose to secure the root while at the same time allowing for the root cells to be accessible to the various chemical treatments ([5]; see Methods). It is possible that some of the [Ca^2+^]_cyt_ transients observed in response to MS application resulted from roots that were not securely anchored to the agarose medium. Alternatively, the prevalence of MS-induced [Ca^2+^]_cyt_ increases in some of our experiments could be due to osmotic changes when the solution was applied. Because agar for imaging roots was poured in advance, it was possible that liquid in some of the plates may have evaporated during storage. This could have led to some plates having an MS solution that was more concentrated than the treatment solution.

To test the possibility that MS-induced [Ca^2+^]_cyt_ changes were due to touch or hypoosmotic shock, we repeated the ATP application experiments using roots that emerged from seeds planted on plates with freshly prepared MS-supplemented agarose. Furthermore, roots were pretreated with MS solution prior to ATP treatment. Under these modified growth and treatment conditions, we found that MS-induced [Ca^2+^]_cyt_ increases were significantly dampened (Figure 3 and Figure 7). Given that [Ca^2+^]_cyt_ changes are responses that can be elicited by a wide range of stimuli, the results described here highlight the need to carefully consider the growth and treatment conditions, and to include appropriate controls when conducting [Ca^2+^]_cyt_ imaging experiments. Based on these observations, it is imperative that fresh plates be prepared prior to planting the reporter lines and that roots be pretreated with the solvent control solution for a few hours to allow seedlings to adapt.

Some studies have shown [Ca^2+^]_cyt_ signals propagate along the longitudinal axis of roots [5,26]. Meanwhile, another study has indicated that [Ca^2+^]_cyt_ signal propagation from the apical into the sub-apical can also occur [27]. The cell type specific GCaMP3 lines described here, particularly those lines in which *GCaMP3* is expressed in inner root cells such as the cortex-, endodermis- and columella-specific lines could shed new insights into the direction of [Ca^2+^]_cyt_ signal propagation within the roots. The MS-induced [Ca^2+^]_cyt_ responses in lines in which *GCaMP3* was expressed in the endodermis and columella were not observed. However, these cell types exhibited [Ca^2+^]_cyt_ increases in response to some of the chemicals, indicating that these [Ca^2+^]_cyt_ changes can be attributed to the chemical itself. In this regard, it is noteworthy that columella-targeted GCaMP3 lines did not exhibit a [Ca^2+^]_cyt_ increase after Al^3+^ treatment while those lines with *GCaMP3* expressed in the endodermis lacked a [Ca^2+^]_cyt_ response to NaCl. With the establishment of response patterns from the cell type specific GCaMP3 lines when exposed to various chemical treatments, investigation into unique aspects of different root tissues/cells, such as signal propagation direction and response intensity, can be more readily explored.

## 4. Materials and Methods

### 4.1. Generation of Cell-Type Specific Promoter GCaMP Lines

Each promoter was amplified from genomic DNA extracted from wild-type *Arabidopsis thaliana* seedlings using the Plant DNAzol Reagent (Invitrogen, Carlsbad, CA, USA) (Table S1). The sequence preceding the start codon for each gene was used in each instance. The resulting fragments were digested with PstI and SalI (New England Biolabs, Ipswich, MA, USA; http://neb.com) and cloned into a modified pCAMBIA1390 vector [32]. *GCaMP3* was amplified from plasmid DNA and digested with XmaI and BstEII (New England Biolabs, Ipswich, MA, USA; http://neb.com) and then cloned behind each promoter construct in pCAMBIA1390. To generate *UBQ10:GCaMP3*, a similar approach was taken. *GCaMP3* was again amplified from plasmid DNA and then digested with EcoRI and SpeI (New England Biolabs, Ipswich, MA, USA; http://neb.com) and cloned behind the *Ubiquitin 10* promoter in a previously described pCAMIA1390 vector [33]. Plasmid containing *GCaMP3* was a gift from Loren Looger (Addgene plasmid # 22692; http://n2t.net/addgene:22692; RRID:Addgene_22692; [34]). *Agrobacterium tumefaciens*-mediated transformation using the floral dip method was used to generate Arabidopsis ecotype Col-0 plants expressing the GCaMP3 constructs [35]. All primers used in this study are listed in Table S2.

### 4.2. Preparation of A. thaliana Seedlings for Imaging

Seeds of the various *A. thaliana* GCaMP3 lines were planted on coverslips coated with a thin layer of MS-supplemented low-melting agarose. This set-up was prepared by pouring 3 mL of autoclaved 0.5% low-melting agarose in 0.5X MS solution (pH 5.7) on 48 × 60 mm No. 1 coverslips (Thermo Scientific, gold seal cover glass reorder no. 3334, Waltham, MA, USA; www.thermoscientific.com) as described in Rincon-Zachary et al. (2010) [5]. Coverslips with polymerized agarose were placed in 9 cm diameter round Petri dishes and stored at 4 °C prior to planting seeds. In another set of experiments, seeds were planted as soon as the agarose polymerized. Petri dishes and coverslips with the planted seeds were kept in 4 °C for 2 days and transferred to a Conviron growth chamber (Controlled Environments Ltd., Winnipeg, MB, Canada) set to 24 °C with 14 h/10 h day/night cycle. Petri dishes with the coverslips were kept vertical to enable the roots to grow down and straight along the surface of the agarose. Seedlings were imaged when primary roots were about 3–4 cm long, which was 5–6 days after transfer to the Conviron.

### 4.3. Validation of GCaMP3 Expression in Root Cell Types

Validation of GCaMP3 expression in the target root cell types was conducted with an inverted Leica TCS SP8-X Confocal Laser Scanning Microscope (Leica Microsystems, Wetzlar, Germany; http://leica-microsystems.com). Seedlings from the Conviron were secured in a horizontal orientation on the stage of the microscope. Seedling roots were illuminated with the 488 nm line of the Argon laser using a 40× (numerical aperture 1.1) water immersion objective and emitted light detected at 510 nm. For some lines, single optical images were acquired at a pixel resolution of 1024 × 1024. For other lines, a Z-series was acquired by capturing 81 images at 0.4 µm intervals and 3-D images were generated using the LAS visualization software of the Leica confocal microscope.

### 4.4. Chemical Treatments and Measurement of Ca^2+^-Dependent GCaMP3 Fluorescence

Stock solutions of 1 M ATP (Sigma-Aldrich, St. Louis, MO, USA; A7699-1G), glutamate (Sigma-Aldrich, St. Louis, MO, USA; G1149-100G), aluminum chloride (Sigma-Aldrich, St. Louis, MO, USA; Cas. No. 7784-13-6) and sodium chloride (J.T. Baker, Phillipsburg, NJ, USA; 3624-19) were made with deionized water and stored at 4 °C prior to use. Working solutions of 1 mM Al^3+^, 1 mM ATP, 1 mM Glu, and 150 mM NaCl were made by adding the appropriate volume of the stock solution in 0.5X MS at pH 5.7. Working solutions were made fresh prior to the imaging experiments.

To measure [Ca^2+^]_cyt_-dependent GCaMP3 fluorescence, images of growing roots were acquired every 1 s for 10 to 15 min using the Leica SP8-X confocal microscope. Images were captured at a scanning speed of 600 MHz and pixel resolution of 512 × 300. Baseline GCaMP3 fluorescence of the roots was first acquired for 1 min after placing seedlings on the stage of the microscope. Twenty µL of the treatment solution was then added on top of the agarose medium where the root was growing using an adjustable volume pipette while imaging continued. From the collected images, the average fluorescence intensity was acquired by marking a rectangular region of interest corresponding to the specific cell type using the rectangular selection tool of the SPX-8 LAS software. GCaMP3 fluorescence (F) values were normalized using the formula $I=\;\frac{\left(F-F0\right)}{F0}$ where F is the fluorescence intensity at some time point and F0 is the lowest fluorescence intensity point for the data set. Normalized fluorescence values were reported as the mean ± SE of at least three independent seedlings per GCaMP3 construct [36]. Another round of intensity video imaging was taken for the 0.5× MS control solution to generate the MS control fluorescence curves.

## Figures and Tables

**Table 1:** Reaction time analysis of [Ca^2+^]_cyt_ response where reaction time is the time elapsed from chemical application to maximum GCaMP3 fluorescence (reaction time ± SE; *n* = sample size; reaction time is in seconds). Statistical analysis using Analysis of Variance (ANOVA) with Tukey Post-Hoc test (*p* ≤ 0.05 for reported significance) was performed for differences in chemical treatments only due to the lack of comparable ROI sections between cell-type lines. Due to limited sample size, significance may indicate a general trend rather than actual significance. ATP = adenosine tri-phosphate; Glu = glutamate; Al^3+^ = aluminum; MS = control solution; ATP:MS = adapted ATP.

Treatment	*UBQ10:GCaMP3*	*PIN2:GCaMP3*	*PRP3:GCaMP3*	*PEP:GCaMP3*	*SCR:GCaMP3*	*ATHB8:GCaMP3*
**ATP ^ab^**	99.3 ± 4.67 (*n* = 3)	120.3 ± 4.3 (*n* = 3)	210.3 ± 26.5 (*n* = 3)	159.0 ± 16.3 (*n* = 6)	141.2 ± 22.6 (*n* = 5)	134.8 ± 8.0 (*n* = 4)
**Glu ^ab^**	100.0 ± 7.1 (*n* = 3)	94.0 ± 4.9 (*n* = 3)	114.0 ± 16.4 (*n* = 3)	145.7 ± 28.0 (*n* = 3)	128.7 ± 17.1 (*n* = 3)	114.7 ± 3.3 (*n* = 3)
**Al^3+ ac^**	196.4 ± 16.5 (*n* = 5)	178.3 ± 37.8 (*n* = 7)	219.0 ± 27.0 (*n* = 6)	154.7 ± 8.0 (*n* = 3)	325.0 ± 94.4 (*n* = 3)	569.0 ± 12.8 (*n* = 3)
**MS ^c^**	174.3 ± 5.6 (*n* = 8)	201.7 ± 33.5 (*n* = 6)	266.2 ± 27.5 (*n* = 5)	217.0 ± 46.6 (*n* = 6)	350.5 ± 77.7 (*n* = 6)	373.8 ± 88.2 (*n* = 6)
**ATP:MS ^c^**	315.3 ± 40.4 (*n* = 3)	404.0 ± 33.7 (*n* = 3)	329.0 ± 95.9 (*n* = 3)	366.0 ± 18.9 (*n* = 3)	424.7 ± 32.9 (*n* = 3)	311.7 ± 9.3 (*n* = 3)

^a,b,c^ indicates significant differences between chemical treatments.

## Supplementary Materials

The following are available online at https://www.mdpi.com/1422-0067/21/17/6385/s1, Supplementary video 1 Time lapse video of [Ca^2+^]_cyt_ changes in the columella cells of *ATHB8:GCaMP3* lines in response to ATP. Red indicates elevated [Ca^2+^]_cyt_. Total elapsed time is 5 min with images captured every 1 s. Video was sped up to 30 frames per second. Supplementary video 2 Time lapse video of [Ca^2+^]_cyt_ changes in the cortex of *PEP:GCaMP3* lines in response to salt. Red indicates elevated [Ca^2+^]_cyt_. Total elapsed time is 10 min with images captured every 1 s. Video was sped up to 60 frames per second. Table S1 Promoters used for development of *GCaMP3* constructs that target to specific cell types in *Arabidopsis thaliana* roots. Table S2 Primer sequences used to generate the *GCaMP3* constructs in *Arabidopsis thaliana*.
