# Supplementary material for: Cell Type-Specific Imaging of Calcium Signaling in Arabidopsis thaliana Seedling Roots Using GCaMP3
Source: Int J Mol Sci. 2020 Sep 2;21(17):6385. doi: 10.3390/ijms21176385 (PMC7503278; doi:10.3390/ijms21176385)
Supplement: Supplementary file 1 [file ijms-21-06385-s001.zip › Supplemental Files ijms-892115/Supplementary Table S1 -Krogman et al.docx]

**Table S1.** Promoters used for development of GCaMP3 constructs that target specific tissue-types in *Arabidopsis thaliana* while also showing expression location in the plant root.

| **Promoter** | **Locus ID** | **Size** | **Expression Location** |
| --- | --- | --- | --- |
| *ATHB8* | At4g32880 | 1.7Kb | Columella |
| *SCR* | At3g54220 | 2.1Kb | Endodermis |
| *PRP3* | At3g62680 | 2Kb | Trichoblast |
| *PIN2* | At5g57090 | 1.4Kb | Epidermis/Cortex |
| *PEP* | At1g09750 | 1.4Kb | Cortex |
| *UBQ10* | At4g05320 | 636Bp | All Tissue Types |
