# Supplementary material for: Cell Type-Specific Imaging of Calcium Signaling in Arabidopsis thaliana Seedling Roots Using GCaMP3
Source: Int J Mol Sci. 2020 Sep 2;21(17):6385. doi: 10.3390/ijms21176385 (PMC7503278; doi:10.3390/ijms21176385)
Supplement: Supplementary file 1 [file ijms-21-06385-s001.zip › Supplemental Files ijms-892115/Supplementary Table S2 -Krogman et al.docx]

**Table S2.** The primer sequences used to generate the GCaMP3 constructs in *Arabidopsis thaliana*.

| **Primer Name** | **Primer Sequence** |
| --- | --- |
| ATHB8F | CATCTGCAGCGGATAAACCAATTTTCAAATGAT |
| ATHB8R | CATGTCGACCTTTGATCCTCTCCGATCTCTCTA |
| SCRF | CATCTGCAGGAGGACTTTGTTTATCAGAAACCT |
| SCRR | CATGTCGACGGAGATTGAAGGGTTGTTGGTCGT |
| PRP3F | CATCTGCAGAGCAAAGACATGAATAAACAAATA |
| PRP3R | CATGTCGACTGCTGAGCGCTTGGCTTGTATATT |
| PIN2F | CATCTGCAGTTGAATCTTTCAATAGTTTCATCC |
| PIN2R | CATGTCGACTTTGATTTACTTTTTCCGGCGAGA |
| PEPF | CATCTGCAGAATGTAAATTAATAAGAGCTACAG |
| PEPR | CATGTCGACGGTTTTGGCTAATGTGATTGTGTA |
| GCaMPF | CATCCCGGGATGGGTTCTCATCATCATCATCA |
| GCaMPR | TACGGTCACCTTACTTCGCTGTCATCATTTGTAC |
| UBQ:GCaMP3F | CATGAATTCATGGGTTCTCATCATCATCATCA |
| UBQ:GCaMP3R | TACACTAGTTTACTTCGCTGTCATCATTTGTAC |
